# Physiological adjustment of pomegranate pericarp responding to sunburn and its underlying molecular mechanisms

**DOI:** 10.1186/s12870-022-03534-8

**Published:** 2022-04-04

**Authors:** Chunyan Liu, Ying Su, Jiyu Li, Botao Jia, Zhen Cao, Gaihua Qin

**Affiliations:** 1 Key Laboratory of Genetic Improvement and Ecophysiology of Horticultural Crops, Institute of Horticulture, Anhui Academy of Agricultural Sciences, Hefei, 230031 China; 2 Key Laboratory of Fruit Quality and Developmental Biology, Anhui Academy of Agricultural Sciences, Hefei, 230031 China

**Keywords:** Sunburn, Metabolite, Genes, Physiological, Pomegranate

## Abstract

**Background:**

Sunburn is common in pomegranate, and sunburned fruits have poor appearance and low marketability. However, the physiological and metabolic responses to sunburn and their underlying molecular mechanisms in pomegranate fruit are little understood. Fruit of sunburn-sensitive cultivar ‘Hongyushizi’ was used to carry out physiological parameter detection and widely-targeted metabolomics and transcriptome study.

**Results:**

Malondialdehyde and relative conductivity increased with the severity of sunburn, which indicated increased membrane injury. Meanwhile, the content of antioxidants (total phenols and flavonoids), which reduce and repair membrane damage, increased and were accompanied by increases in total antioxidant capacity. In sunburned fruits compared with controls, 129 metabolites changed (including naringenin, pelargonidin and kaempferol) and 447 differentially expressed genes including *CHI* (*Pgr25966.1*), *F3′5′H* (*Pgr26644.1*), and *CHS (Pgr005566.1)* may have contributed to these changes. Transcription factors, such as *NAC 5* (*Pgr008725.1*), *MYB 93* (*Pgr001791.1*), and *MYB 111* (*Pgr027973.1*) may be involved in phenylpropanoid and flavonoid biosynthesis by regulating the *CHI*, *F3′5′H*, and *CHS* etc.

**Conclusions:**

These findings provide insight into the sunburn mechanisms of pomegranate, and also into the genetic improvement of fruit sunburn.

**Supplementary Information:**

The online version contains supplementary material available at 10.1186/s12870-022-03534-8.

## Background

Pomegranate (*Punica granatum* L.) is an ancient crop that originated from Central Asia and is now widely grown in subtropical and tropical areas [1]. This tree species is well adapted to marginal lands and arid soils. In China, pomegranate is mainly grown in the Henan, Anhui, Shandong, and Shanxi provinces, where extreme summer air temperatures reach 40 °C and fruit sunburn is common [2]. Pomegranate is terminal-bearing plant with thin branches. With the increase of fruit weight, the thin branches gradually bend, and the fruit becomes more vulnerable to sunburn [3]. Because pomegranate fruit grow through summer and mature in autumn, they are exposed to intense solar radiation and high temperatures throughout the summer, leading to sunburn on the exposed surface of the pericarp. Sunburn usually reduces the harvest of pomegranate fruit by 30% [3].

Sunburn is a physiological disorder that frequently occurs in fruit growing in areas with warm climates, resulting from an excess of heat and light irradiance [4]. The damage from sunburn to plants mainly includes inhibition of photosynthesis, cell membrane damage, senescence, and cell death [5, 6]. The main cause of sunburn is thought to be an increase in production of reactive oxygen species (ROS), which causes oxidative damage due to the incapacity of the fruit to recover from stress [6]. Plants have evolved efficient regulatory systems for perceiving and responding to high temperature/solar irradiance stress [6, 7]. Antioxidant enzyme and non-enzymatic antioxidants, such as ascorbic acid (AsA), glutathione (GSH), and flavonoids, work in concert with antioxidant enzymes to cope with the intracellular generation of ROS, and strengthen the responses to abiotic/biotic environmental stressors [8, 9].

As sunburn browning occurs or increases in severity, the concentration of antioxidant metabolites increases [6]. Heating apple leaves from 28 to 40 °C increased the content of antioxidant compounds, but after 4 h at 40 °C the antioxidant compounds decreased [10]. Furthermore, in apple peel, antioxidants were up-regulated in response to the increased ROS generated by exposure to high light and temperature [11]. Flavonoids, together with carotenoids and anthocyanins, enhance light absorption in the UV and blue regions of the spectrum and act as scavengers of ROS molecules [12]. Given the detoxifying activity of AsA on O^2−^, AsA could be further required for preventing sunburn browning of fruit [13]. However, fruit with severe sunburn showed a lower total phenol content and total antioxidant activity than undamaged fruit [14]. Responses to sunburn have been investigated in sunburn-susceptible fruits, such as apple and grape [15, 16]. However, knowledge of the physiological changes as well as the underlying genetic mechanism response to sunburn in pomegranate is lacking.

To address these issues, physiological changes in pomegranate pericarp with different degrees of sunburn were studied. Then un-targeted metabolome and transcriptome analyses were conducted for severe sunburned pericarp and healthy pericarp. These results provided functional components of pomegranate fruits responding to sunburn and the potential genes underlying the metabolites that showed different accumulations.

## Results

### Physiological responses of pomegranate pericarp to sunburn

**Fig. 1:**
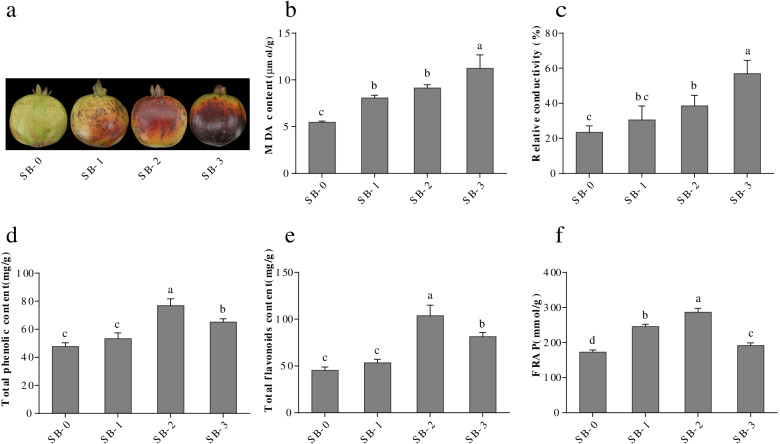
**a** The degree of sunburn of ‘Hongyushizi’ pomegranate. SB-0, no sunburn; SB-1, mild sunburn; SB-2, moderate sunburn; SB-3, severe sunburn. **b** MDA content in pomegranate pericarp with four degrees of sunburn. **c** Relative conductivity in pomegranate pericarp with four degrees of sunburn. **d** The total phenolic content in pomegranate pericarp with four degrees of sunburn. **e** The flavonoid content in pomegranate pericarp with four degrees of sunburn. **f** The antioxidant activity in pomegranate pericarp with four degrees of sunburn. The small letters on different columns indicate significant differences

To investigate the physiological response of pomegranate pericarp to sunburn stress, three grades of sunburned pomegranate pericarp and the control were analyzed. In biomembranes, MDA is one of end products of lipid peroxidation. Pomegranate pericarp suffering from sunburn showed a 47.9–105.8% increase in MDA content compared with control, which reflected increased membrane injury (Fig. 1b). Additionally, under sunburn, pomegranate pericarp showed a 30.4–143.1% increase in relative conductivity, indicating severe membrane integrity impairment (Fig. 1c). Sunburn induced an increase in the content of antioxidants to reduce and repair the damage caused by ROS. Compared with the control, total phenol content increased by 11.6–60.5% and flavonoid content increased by 17.0–114.9%, whereas total phenols and flavonoids were down-regulated significantly in SB-3 compared with SB-2 (Fig. 1d, e). This was accompanied by increased antioxidants, with total antioxidant capacity (FRAP) increasing 42.1–108.3% compared with control, whereas the total antioxidant capacity was down-regulated in SB-3 compared with SB-2 and SB-1 (Fig. 1f). In short, the SB-3 pomegranate had the most severe membrane injury and significantly increased antioxidants and total antioxidant capacity. Thus, pomegranate pericarps with SB-0 (control) and SB-3 (sunburn treatment) were selected for further metabolomic and molecular studies in response to sunburn stress.

### Metabolome of pomegranate pericarp responding to sunburn

**Fig. 2:**
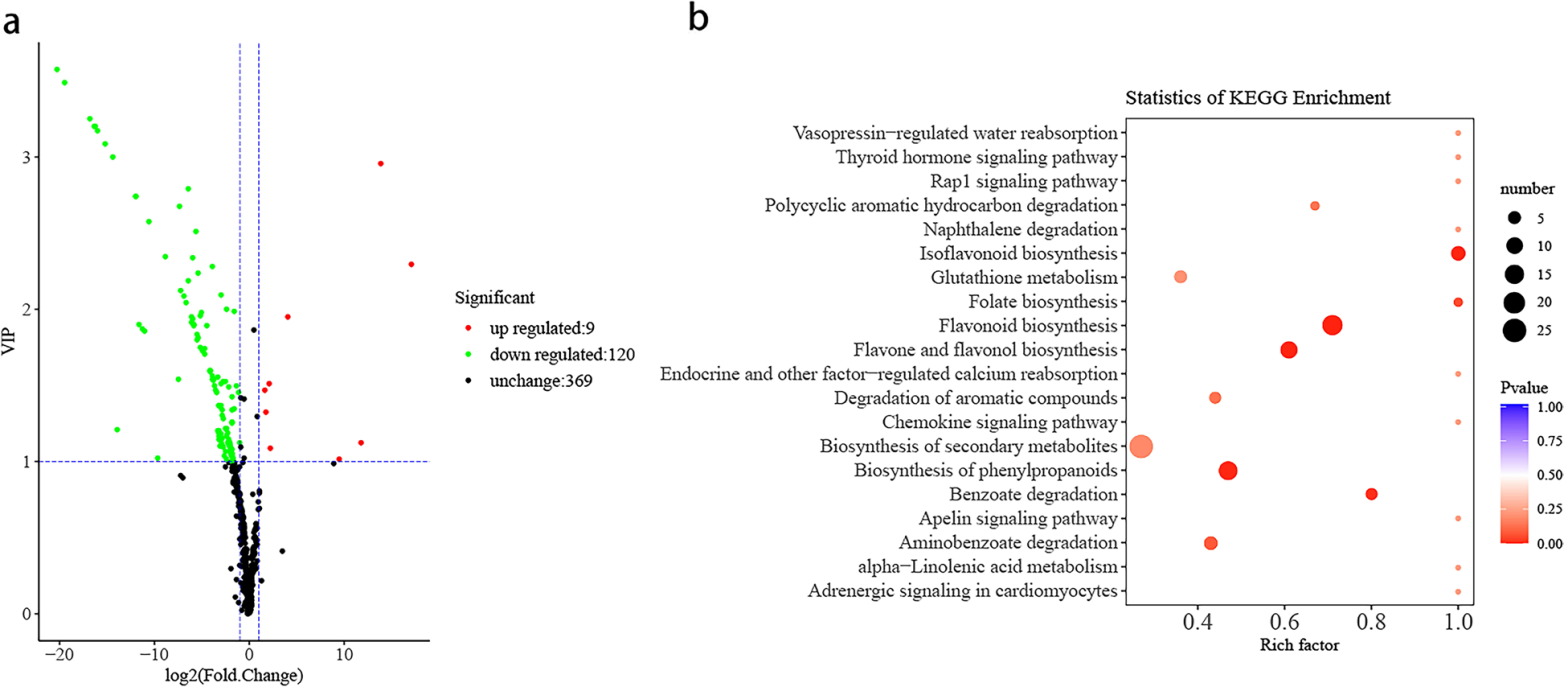
**a** Volcano plot of DAMs in SB-0 vs. SB-3. Red dots are up-regulated metabolites, green dots are down-regulated metabolites, and black dots are unchanged metabolites. **b** Bubble diagram of KEGG pathway enrichment analysis based on the DAMs in SB-0 vs SB-3

To further investigate the metabolomic response to sunburn, we conducted metabolic profiling analysis on SB-3 and SB-0. A total of 498 metabolites were identified in pomegranate pericarps. We found that 129 differentially accumulated metabolites (DAMs) including flavonoids, lipids, organic acids, amino acids, amino acid derivatives, and vitamins in SB-3 compared to SB-0 (Supplementary Table 1, Fig. 2a).

Then we conducted a KEGG pathway enrichment analysis of DAMs to focus on the pathways responding to sunburn. We found that five pathways were enriched: biosynthesis of secondary metabolites (ko01110), flavonoid biosynthesis pathways (ko00941), biosynthesis of phenylpropanoids pathways (ko01061), flavone and flavonol biosynthesis pathways (ko00944), and isoflavonoid biosynthesis pathways (ko00943) (Fig. 2b). Furthermore, we found that most DAMs decreased in SB-3 compared to SB-0, while nine DAMs including amino acid derivatives (L-γ-glutamylcysteine, L-cysteinyl glycine, and glutathione reduced form), a flavone (tricin 5-O-hexoside), an organic acid (hydroxy-3-methoxymandelate), a vitamin (L-ascorbate), an anthocyanin (delphinidin 3-O-rutinoside), a coumarin (7-hydroxy-5-methoxycoumarin), and a proanthocyanidin (procyanidin A3) were up-regulated. In the flavonoid biosynthesis pathway, 16 DAMs were down-regulated. In addition to biosynthesis of flavonoids, the phenylpropanoid metabolic branches also contributed to the production of coumarins and the non-flavonoid product scopoletin (7-hydroxy-5-methoxycoumarin, pme2993), which is a coumarin and an antioxidant, were markedly up-regulated [17]. Caffeic acid (pme0303) and p-coumaryl alcohol (pme3305) were down-regulated in the sunburned pomegranate pericarp.

### Differentially expressed genes in sunburned pomegranate pericarp

**Fig. 3:**
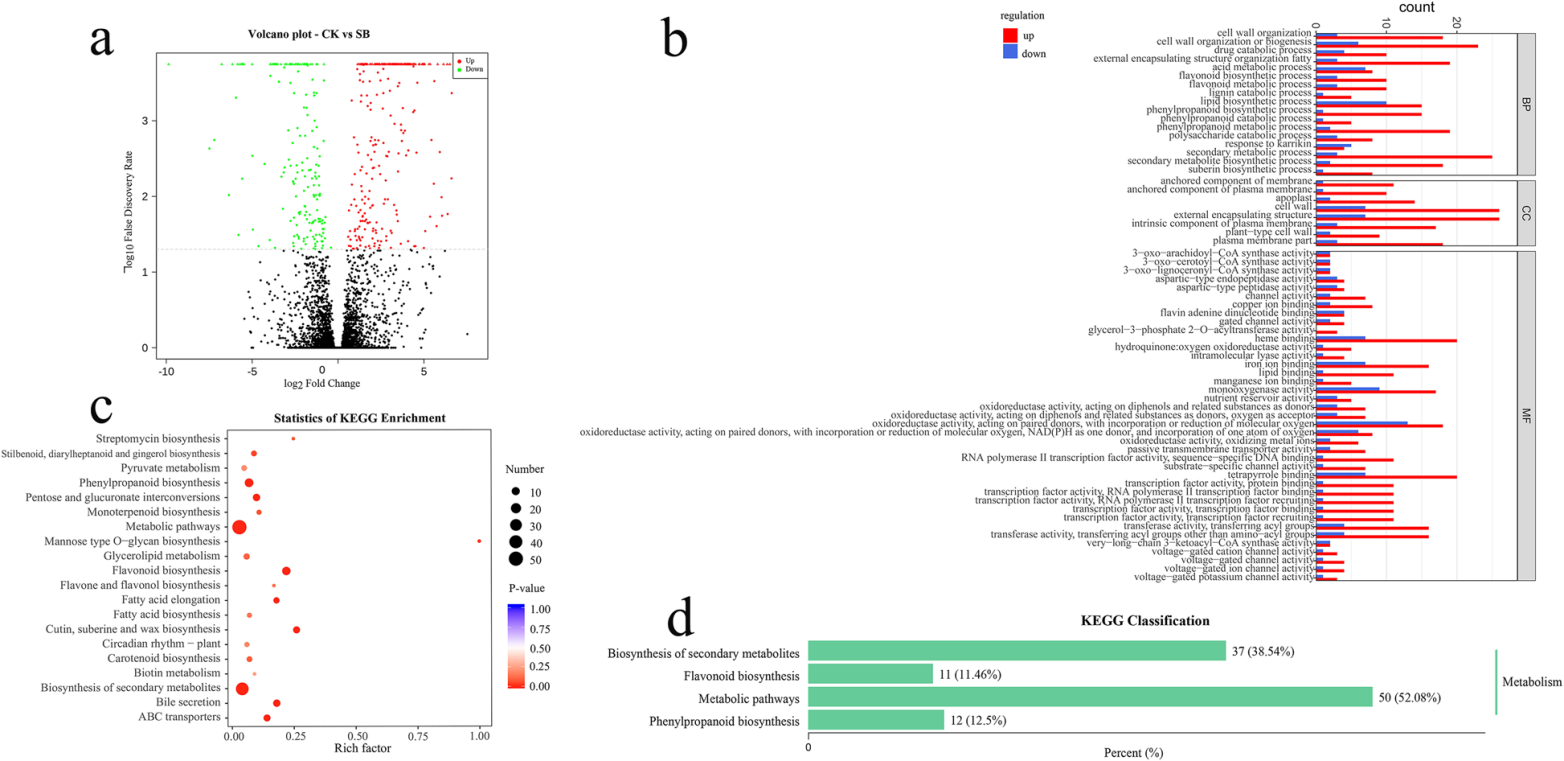
**a** Volcano plot of DEGs in SB-0 vs SB-3. **b** Functional GO classification of DEGs. The enriched GO terms are shown on the y-axis. The counts of the up-regulated and down-regulated DEGs are shown on the x-axis. Directed acyclic graphs of three main categories are displayed in the thumbnail view. **c** Bubble diagram of the KEGG pathway enrichment analysis based on the DEGs in SB-0 vs. SB-3. **d** KEGG annotation results of the first four pathways with most DEGs. The abscissa represents the proportion of genes annotated to the pathway to the total number of annotated genes, and the ordinate represents the name of the pathway

To understand the genetic mechanisms underlying the different accumulations of metabolites in response to sunburn, we conducted transcriptome analysis on sunburned and control pericarps. A total of 40.29–52.97 million clean reads was obtained through sequencing of the cDNA libraries after stringent quality checks and data cleanup (Supplementary Table 2). The alignment showed that more than 86.47% of clean reads were mapped to the reference genome. Screening of differentially expressed genes (DEGs) showed that 277 genes were up-regulated and 170 were down-regulated with sunburn (Fig. 3a, Supplementary Table 3). Then we conducted GO functional enrichment analysis of all DEGs, and they were divided into three categories: biological processes, cell components, and molecular functions (Fig. 3b). The GO analysis showed that the most DEGs were grouped in the cell wall external encapsulating structure, and oxidoreductase activity, cell wall organization or biogenesis, and secondary metabolic process. To further understand the biological metabolic pathways and the candidate genes contributing to metabolite accumulations, we conducted KEGG pathway enrichment analysis for DEGs. The DEGs were assigned to 85 metabolic pathways (*P* < 0.05), of which the significantly enriched pathways included metabolic pathways (ko01100, 50 genes), biosynthesis of secondary metabolites (ko01110, 37 genes), phenylpropanoid biosynthesis (ko00940, 12 genes), and flavonoid biosynthesis (ko00941, 11 genes) (Fig. 3c, d).

**Fig. 4:**
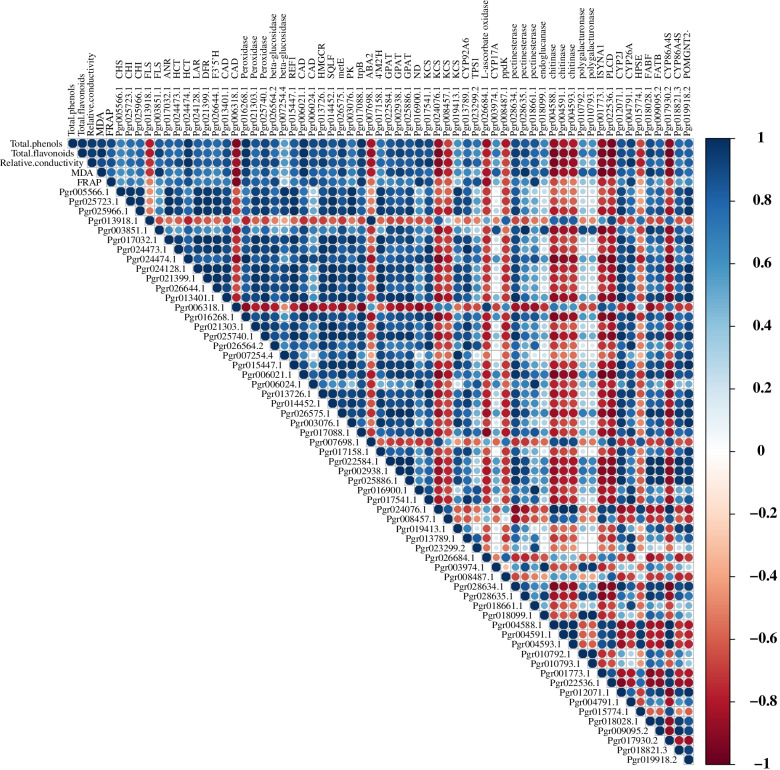
Correlation analysis for DEGs involved in metabolic pathways, biosynthesis of secondary metabolites, phenylpropanoid biosynthesis, and flavonoid biosynthesis and physiological indexes (total phenols, total flavonoids, relative conductivity, MDA, FRAP). *CHS* chalcone synthase, *CHI* chalcone isomerase, *FLS* flavonol synthase, *ANR* anthocyanidin reductase, *HCT* shikimate O-hydroxycinnamoyltransferase, *LAR* leucoanthocyanidin reductase, *DFR* bifunctional dihydroflavonol 4-reductase/flavanone 4-reductase, *F3′5’H* flavonoid 3',5'-hydroxylase, *REF1* coniferyl-aldehyde dehydrogenase, *CAD* cinnamyl-alcohol dehydrogenase, *HMGCR* hydroxymethylglutaryl-CoA reductase, *SQLE* squalene monooxygenase, *metE* 5-methyltetrahydropteroyltriglutamate-homocysteine methyltransferase, *PK* pyruvate kinase, *trpB* tryptophan synthase beta chain, *ABA2* xanthoxin dehydrogenase, *4’M2’H* isoflavone/4'-methoxyisoflavone 2'-hydroxylase, *GPAT* glycerol-3-phosphate acyltransferase, ND( +)-neomenthol dehydrogenase, *KCS* 3-ketoacyl-CoA synthase, *CYP92A6* typhasterol/6-deoxotyphasterol 2alpha-hydroxylase, *TPS1* ( +)-alpha-terpineol/(4S)-limonene synthase, *CYP17A* steroid 17 alpha-monooxygenase/17 alpha-hydroxyprogesterone deacetylase, ppdK pyruvate, orthophosphate dikinase, *ISYNA1* myo-inositol-1-phosphate synthase, *PLCD* phosphatidylinositol phospholipase C, delta, *CYP2J* cytochrome P450 family 2 subfamily J, *CYP26A* cytochrome P450 family 26 subfamily A, *HPSE* heparanase, FABF 3-oxoacyl-[acyl-carrier-protein] synthase II, *FATB* fatty acyl-ACP thioesterase B, *FAOH* fatty acid omega-hydroxylase, *POMGNT2* protein O-mannose beta-1,4-N-acetylglucosaminyltransferase

To understand which genes contribute most to sunburn, correlation between DEGs and physiological indexes was carried out (Fig. 4). Seventeen of 23 DEGs in the phenylpropanoid and flavonoid biosynthesis pathways showed correlation with physiological response of pomegranate, particularly *Pgr024474.1* encoding *HCT*, *Pgr016268.1* encoding *peroxidase*, *Pgr006021.1* encoding *CAD* had high positive correlation coefficient (*R* > 0.83 and *P* < 0.05) between its expression pattern and the physiological response. However, the expression pattern of *Pgr006318.1* encoding *CAD* had a negative correlation with the physiological index. These results indicate the phenylpropanoid and flavonoid biosynthesis pathways contribute most to pomegranate sunburn.

### Comprehensive analysis of transcripts and metabolites responding to sunburn

**Fig. 5:**
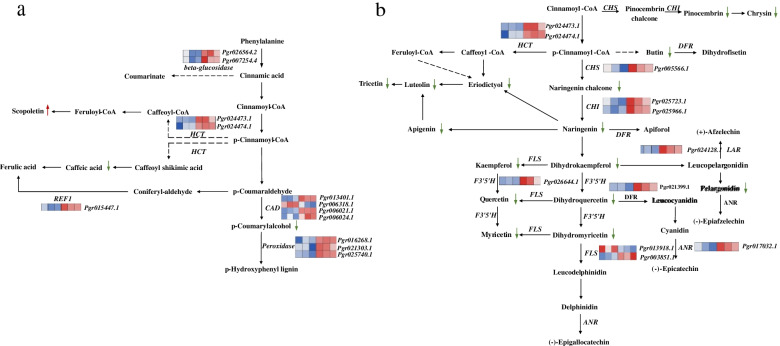
The phenylpropanoid and flavonoid biosynthesis pathways in pomegranate pericarps under sunburn stress. The biosynthetic pathway of phenylpropanoid and flavonoid referring to the KEGG database (https://www.kegg.jp/kegg-bin/show_pathway?map00940, https://www.kegg.jp/kegg-bin/show_pathway?map00941) [46]. Expression patterns of genes in the phenylpropanoid and flavonoid biosynthesis pathways shown as a heatmap. The three on the left represent healthy fruit, and the three on the right represent sunburned fruit. The red indicates higher and the blue indicates lower. The compounds up-regulated in the metabolome analysis are marked by red arrows, and the compounds down-regulated are marked by green arrows

**Fig. 6:**
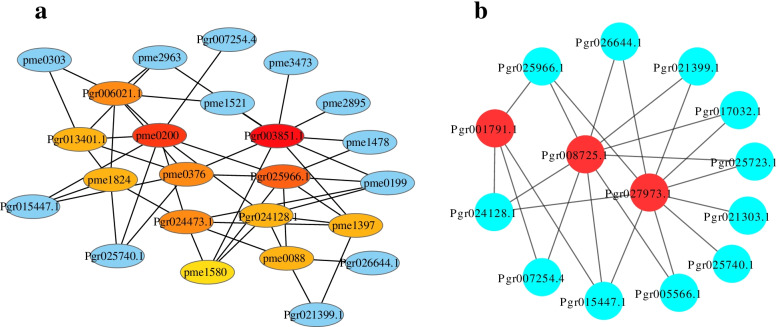
**a** Association network analysis of structural genes and metabolites of phenylpropanoid and flavonoid biosynthesis pathways. The darker nodes indicate higher reliability. Edge lines represent the relationship between transcripts and compounds. **b** Co-expression analysis of genes and transcription factors related to the phenylpropanoid and flavonoid pathways. Genes with correlation coefficients > 0.95 are shown. Red circles represent transcription factors and blue circles represent structural genes

To focus on the candidate genes contributing to DAMs responding to sunburn, we performed comprehensive analysis of the DEGs and DAMs in the phenylpropanoid and flavonoid biosynthesis pathways (Fig. 5). Twelve DEGs were strongly correlated (*R* > 0.95 and *P* < 0.05) with 18 DAMs following sunburn in the phenylpropanoid and flavonoid biosynthetic pathways. The top 20 nodes are shown in the network diagram (Fig. 6a). There was a significant correlation between the expression of the *Pgr003851.1* (*FLS*) and naringenin (pme0376), a naturally occurring flavanone, and pelargonidin (pme1397), a well-known natural anthocyanidin. Kaempferol (pme0200), an anti-inflammatory flavonoid commonly found in plants, was significantly correlated with the expression of genes *Pgr25966.1* (*CHI*), *Pgr13401.1* (*CAD*), *Pgr006021.1* (*CAD*), and *Pgr024473.1* (*HCT*).

Additionally, there were 29 TFs, including *MYB*, *NAC*, *bHLH*, and *HY* TFs, showing different expression in SB-3 compared to SB-0 (Supplementary Table 4). For example, *Pgr001791.1* encoding *MYB93*, a gene negatively regulating genes involving in anthocyanin and proanthocyanidin biosynthesis [18], was 20 times up-regulated in sunburned fruits compared to the control. Gene *Pgr027973.1* encoding *MYB111*, which activates the expression of genes involved in flavonol synthesis [19], had high expression following sunburn. There are reports on TFs playing a vital role in the regulation of target genes under abiotic and biotic stress in plants [20, 21]. This implied that the TFs played roles in regulating genes involved in metabolite accumulation responding to sunburn.

Then, we conducted co-expression analysis of differentially expressed TFs and genes encoding enzymes that participate in phenylpropanoid and flavonoid biosynthesis and found that *Pgr027973.1*, encoding *MYB111*, *Pgr008725.1*, encoding *NAC5*, and *Pgr001791.1*, encoding *MYB93*, were co-expressed with genes involved in phenylpropanoid and flavonoid biosynthesis (*R* > 0.95) (Fig. 6b). Thus, we conclude that *Pgr027973.1*, *Pgr008725.1*, and *Pgr0012667.1* may respond to sunburn by regulating genes involving in phenylpropanoid and flavonoid biosynthesis.

### Confirmation of transcriptome data using qRT-PCR

**Fig. 7:**
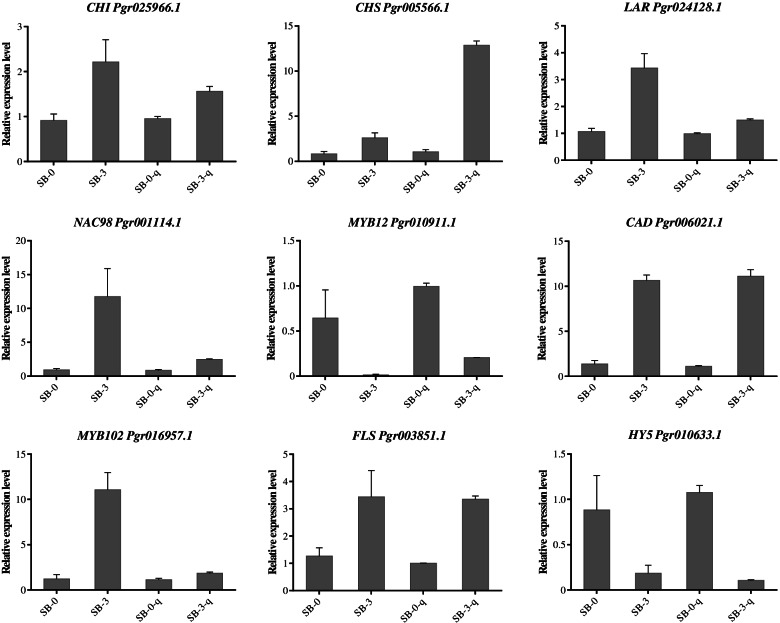
Expression of representative genes in healthy (SB-0) and severely sunburned (SB-3) pomegranate pericarps validated by qRT-PCR. SB-0-q and SB-3-q represent qPCR analysis; SB-0 and SB-3 represent the FPKM value of RNA-seq

Five genes involved in phenylpropanoid and flavonoid biosynthesis pathways and four TFs were selected to validate the RNA-seq results using qRT-PCR (Fig. 7). The results showed that the relative expressions of DEGs were accordant with the transcript accumulations of DEGs in RNA-seq study, indicating the reliability of RNA-seq results.

## Discussion

High temperature and excess light are a general source of oxidative stress, leading to plant cell metabolism disorders and producing high levels of ROS in the plant. Excessive accumulation of ROS in cells causes oxidative damage to membranes (lipid peroxidation), and triggers the production of free radical scavengers of enzymatic (CAT, APX, SOD, peroxidase) or non-enzymatic systems in response to oxidative damage [6]. In sunburned pomegranate pericarps, the increased MDA content and relative electrical conductivity may be the result of increased membrane injury. Phenols and flavonoids are two important metabolite types in the antioxidant systems of plants. Phenolic compounds accumulated in the skin of apple fruits exposed to sunlight to protect the fruit from stress [21]. Polyphenols possess ideal structural chemistry for free radical scavenging and they have been shown to be more effective antioxidants in vitro than tocopherols and AsA [22]. Flavonoids may be used as scavengers of ROS and can absorb the most energetic short solar wavelengths [23, 24]. Previous studies in *Arabidopsis* have shown that the accumulation of flavonoids, which results in radical scavenging activity in vitro, leads to the enhancement of oxidative and drought tolerance in vivo [24]. In pomegranate, the total phenol and total flavonoid contents increased with increased sunburn, which may be used to reduce and repair the damage caused by ROS. However, the total phenols and flavonoids decreased significantly in pomegranate pericarps with extremely severe sunburn. This indicated the response of antioxidants could be divided into two phases during the stress. The first stage was associated with protection by ROS scavenging and the inhibition of lipid peroxidation and may be related to moderate stress. In the second phase, pomegranate underwent severe stress, their antioxidant degradation exceeded synthesis and so their content decreased. The total phenolic content and antioxidant activity of pomegranate fruit with severe sunburn were lower than those of undamaged fruit [14]. This mechanism can also be expanded to other stress [25] and antioxidants [26].

In addition to total phenols and flavonoids, AsA and GSH also play a crucial role in plant survival under abiotic stresses [5]. It was demonstrated that the contents of antioxidants AsA and GSH tend to decrease under severe short-term light and/or temperature stress [27]. However, the antioxidant content will gradually increase after long-term exposure to adverse light and temperature conditions. This increase may be due to the extended stress (e.g., days or weeks), leading to acclimated, long-term responses. Acclimation is a process in which plants respond to environmental changes (such as light and temperature conditions) so that they can maintain a steady state under diverse environmental conditions [28]. It was confirmed that the concentrations of AsA and GSH increased with increasing severity of sunburn browning [29]. We found a similar phenomenon in pomegranate pericarps with severe sunburn.

To cope with sunburn stress, plants have evolved ways to alter their metabolism and reconfigure metabolic networks. This response occurs at the primary metabolic level after short-term sunburn stress, which then trigger elaborate changes in the production of functional secondary metabolites that can protect against sunburn stress [30]. Correlation between DEGs and physiological index indicate sunburn alters the phenylpropanoid and flavonoid pathway most, triggering a marked accumulation of phenols, which may help alleviate the damaging effects of sunburn stress. Similar studies have showed UV-B irradiation alters the phenylpropanoid and flavonoid pathway in Chinese liquorice (*Glycyrrhiza uralensis*) to protect against UV-B damage [31]. Following sunburn, we found that genes involved in phenylpropanoid and flavonoid biosynthesis were activated and their related metabolite profiles were significantly changed. The DAMs of the phenylpropanoid and flavonoid pathways in SB-3 were down-regulated except for scopoletin. This indicated a higher flux through the coumarin compared with the flavonoid pathway in the sunburned pericarp of pomegranate fruit, which may result in increased products, such as scopoletin, to scavenge ROS [32].

In the present study, key genes encoding the enzymes involved in phenylpropanoid and flavonoid biosynthesis (e.g., *CHI*, *CHS*, *LAR*, and *F3′5′H*) were up-regulated in the pomegranate pericarp following sunburn. Several TFs, including *NAC* and *MYB* TFs, that were activated in pericarp of pomegranate may bind to the promoter regions of key genes encoding the enzymes and play key roles in regulation of DAMs under sunburn. The *NAC* family is one of the largest gene families in the plant genome and numerous studies have examined the involvement of several types of N*ACs* in abiotic stress response [33, 34]. The ANAC078 protein has been found to be associated with the induction of genes related to flavonoid biosynthesis, leading to the accumulation of anthocyanins in response to high-light stress [34]. The stress-responsive protein OsNAC5 has been reported as the transcriptional activator that enhances stress tolerance by up-regulating the expression of stress-inducible rice genes [35]. In the present study, *NAC5* was remarkably accumulated and was associated with the genes (*F′3′5′H*, *LAR*, *CHI*, *CHS*, *DFR*, *ANR*, and *CHS*) critically involved in phenylpropanoid and flavonoid biosynthesis, suggesting a vital role in metabolite accumulation with sunburn. The R2R3-MYB subfamily is the largest group of the plant *MYB* family [36]. Members of the R2R3-MYB family of TFs have been demonstrated to be transcriptional regulators of structural genes of flavonoid biosynthesis [37]. For example, *MYB111* in turnip was regulated by different light spectra, suggesting the role of *MYB111* in response to light stress [38]. The TF *AtMYB111* in Arabidopsis regulates flavonol synthesis and accumulation by activating the expression of genes related to flavonol synthesis (*CHS*, *CHI*, *F3H*, and *FLS1*) [19]. In the present study, the induced *MYB111* promoted expression of *CHI*, *F3′5’H*, *LAR*, *REF*, and beta-glucosidase involved in phenylpropanoid and flavonoid biosynthesis. *MYB57*, which has high homology to *MYB93*, contributes to the negative regulation of anthocyanin and proanthocyanidin biosynthesis in poplar [18]. These findings suggest that *MYB93* may act as a transcriptional repressor, inhibiting the genes expression (*CHI*, *LAR*, *beta-glucosidase*, and *REF*), thereby reducing flavonoid levels in response to sunburn. The exact function of these candidate TFs needs further verification.

## Conclusions

**Fig. 8:**
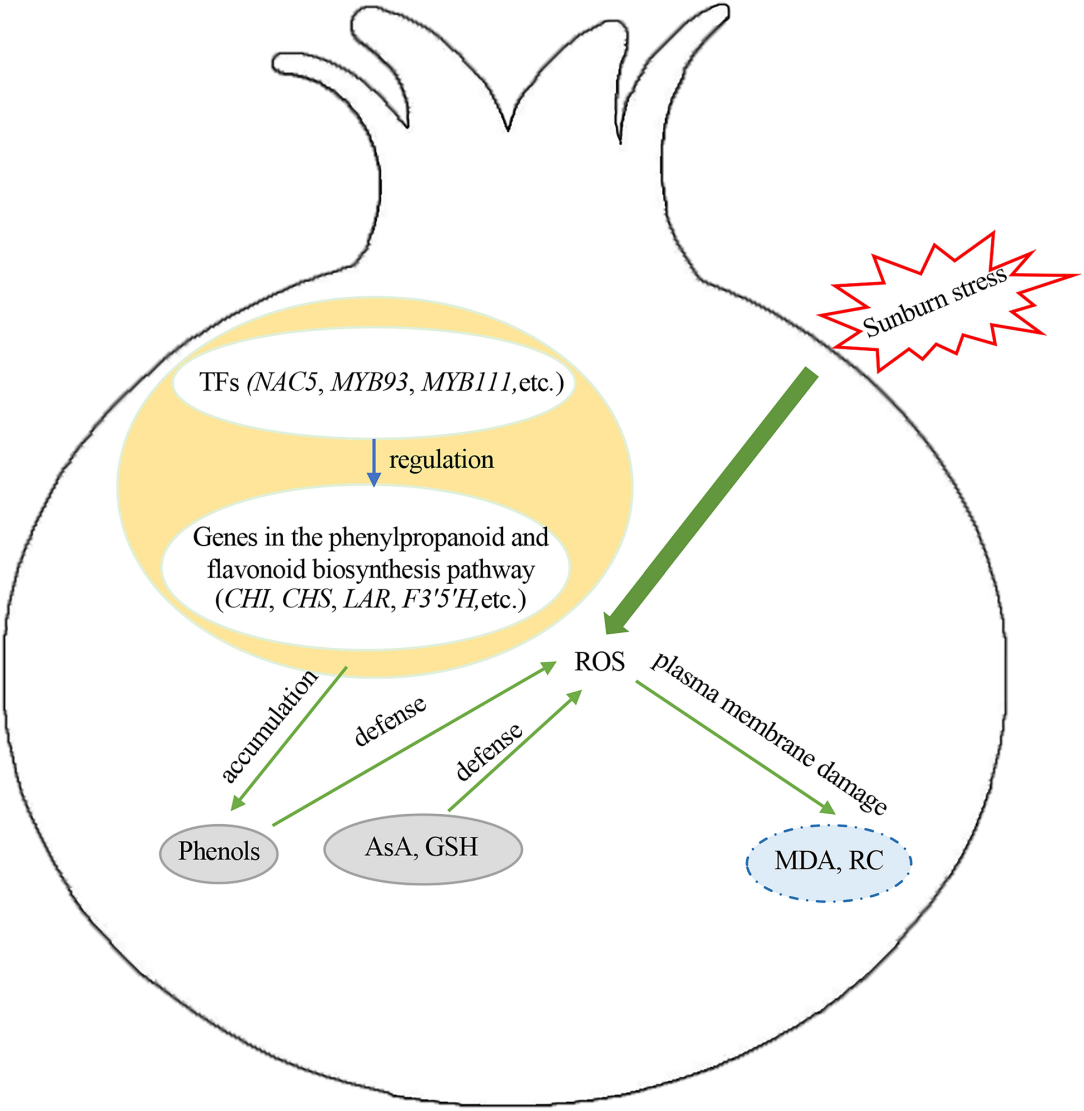
A proposed model of sunburn response mechanisms of pomegranate. *MDA* malondialdehyde, *RC* relative conductivity, *AsA* ascorbic acid, *GSH* glutathione

A proposed model of sunburn response mechanisms of pomegranate was showed in Fig. 8. Sunburn is a physiological disorder resulting from an excess of heat and/or light irradiance. Sunburn stress induced an increase in the content of antioxidants (total phenols, flavonoids, AsA, and GSH); therefore, the total antioxidant capacity increased to reduce and repair the membrane damage. However, as the severity of sunburn increased, the flavonoids tended to decrease and most metabolites in the phenylpropanoid and flavonoid biosynthesis pathways were down-regulated. The key genes encoding the enzymes involved in phenylpropanoid and flavonoid biosynthesis (e.g., *CHI*, *CHS*, *LAR*, and *F3′5′H*) and TFs (e.g., *NAC5*, *MYB93*, and *MYB111*) that were activated in pomegranate pericarp may contribute to the DAMs following sunburn. These findings not only provide insight into the sunburn response mechanisms of pomegranate, but also into genetic improvement of fruit sunburn.

## Methods

### Plant materials

Ripe fruits of *P. granatum* L. ‘Hongyushizi’, a sunburn-sensitive cultivar, were selected to study the sunburn response. *P. granatum* L. ‘Hongyushizi’ is a fruit crop of economic importance and passed the approval of the Anhui Provincial Forest Variety Approval Committee in 2003. Pomegranate ‘Hongyushizi’ were harvested from the Gangji Eco-agricultural Demonstration site, Anhui Academy of Agricultural Sciences, Hefei, China, and the sampling permissions were obtained. We declare that the pomegranate materials in this study comply with institutional, national, and international guidelines for the collection and cultivation of any plant materials. Pomegranate fruit was sorted into four grades according to sunburn browning: no sunburn (SB-0, control); slight sunburn (SB-1, pericarps having dark tan spot); medium sunburn (SB-2, pericarps having sunburn browning); and severe sunburn (SB-3, pericarps having necrosis) (Fig. 1a). Pericarp tissue about 10 mm thick and ± 10 g was dissected from fruit with different grades of sunburn, and then immediately frozen in liquid nitrogen and stored at − 80 °C until use. Three independently replicated experiments with five fruits each were executed. Pomegranate pericarps of four sunburn degrees were used to study the physiological responses to sunburn. Metabolome and transcriptome analyses were conducted on samples rated SB-0 and SB-3.

### Measurements of physiological characteristics and statistical analysis

Malondialdehyde (MDA) levels were determined according to the protocol described by Heath and Packer with modifications [39]. In brief, 7 mL of 10% trichloroacetic acid was added to 0.7 g pomegranate pericarp. The homogenate was centrifuged at 4,000 × g for 10 min. Then, 2 mL of centrifuged supernatant was taken (2 mL of distilled water as control), 2 mL 0.6% thiobarbituric acid (TBA) was added, and the mixture was reacted in a boiling water bath for 15 min. The mixture was then centrifuged after cooling rapidly (10,000 × g for 5 min). The supernatant was used to determine the extinction at 532 and 600 nm (with 0.6% TBA or control as reference). The amount of MDA was calculated by using an extinction coefficient of 155 mM^−1^ cm^−l^.

Electrical conductivity was determined according to the soaking method described by Chen et al. [40]. The total phenol content in the pericarp was determined using a modified Folin–Ciocalteu method [41]. In brief, 100 µL of the prepared sample solution was mixed with 0.5 mL of Folin–Ciocalteu reagent and 1.5 mL of 20% sodium carbonate. The volume was adjusted to 10 mL with distilled water, mixed evenly, and incubated in a water bath for 15 min at 100 °C. The absorbance of the solution was measured against a blank at 765 nm with ultraviolet–visible spectrophotometer. Gallic acid was used as a standard and the total phenols were expressed as mg/g gallic acid equivalent.

The flavonoid content was determined according to the method previously described by Jia with slight modifications [42]. Firstly, 200 μL of extract was placed in a 10 mL volumetric flask. Then, 0.3 mL of 5% NaNO_2_ solution was added, followed by the addition of 0.3 mL of 10% AlCl_3_ (1:10) 6 min later. After 6 min, 1 mL of 1 mol/L NaOH was added and the total was made up to 10 mL with distilled water. The absorbance of the fully mixed solution was measured at 510 nm. Rutin was used as the standard for the calibration curve. According to the calibration curve, the flavonoids content was calculated.

Antioxidant activity (FRAP) was determined according to a procedure adopted from Benzie and Strain [43]. The FRAP reagent contained 2.5 mL of a 2,4,6-tris (2-pyridyl)-s-triazine solution (10 mmol/L) in hydrochloric acid (40 mmol/L) and 2.5 mL of a FeCl_3_ solution (20 mmol/L) blended with 25 mL of an acetate buffer (0.3 mol/L, pH 3.6). FRAP reagent was then warmed to 37 °C. Aliquots of 10 μL diluted sample were mixed with 1.8 mL of FRAP reagent, and then 200 μL of distilled water was added. The absorbance of the reaction mixture was measured at 593 nm. The results were expressed as the concentration of antioxidants with a ferric reducing ability equivalent to that of 1 mmol/L FeSO_4_. Adequate dilation was needed if the FRAP value obtained was greater than the linear range of the standard curve.

Data were analyzed using ANOVA in SPSS Version 16.0 (Chicago, IL, USA). Differences in means were assessed using the least significant difference (LSD) test (*P* < *0.05*).

### Metabolites extraction, identification, and quantification

In order to better separate the metabolites, the samples were freeze-dried and ground into fine powder. The freeze-dried pericarp tissue was crushed using a mixer mill (MM 400, Retsch, Haan, Germany) with a zirconia bead for 1.5 min at 30 Hz. One hundred milligrams of powder were weighed and extracted overnight at 4 °C with 1.0 mL of 70% aqueous methanol. Following centrifugation at 10,000 g for 10 min, the extracted supernatants were absorbed (CNWBOND Carbon-GCB SPE Cartridge, 250 mg, 3 mL; ANPEL, Shanghai, China, www.anpel.com.cn/cnw) and filtered (SCAA-104, 0.22 µm pore size; ANPEL, Shanghai, China, http://www.anpel.com.cn/) before liquid chromatography-tandem mass spectrometry (LC–MS/MS) analysis [44].

Quality control samples (QC) were prepared by mixing sample extracts and used to analyze the repeatability of samples under the same treatment methods. During the instrumental analyses, a quality control sample was inserted into every ten tests and QC samples were analyzed to monitor the repeatability of the analysis. The sample extracts were analyzed using a LC–ESI–MS/MS system (HPLC, Shim-pack UFLC Shimadzu CBM30A system, ww.shimadzu.com.cn/; MS, Applied Biosystems 6500 QTRAP, www.appliedbiosystems.com.cn/). The HPLC analytical conditions were as follow: (1) column, Waters ACQUITY UPLC HSS T3 C18 (1.8 μm, 2.1 mm × 100 mm); (2) solvent system, water (0.04% acetic acid):acetonitrile (0.04% acetic acid); (3) gradient program, 95:5 V/V at 0 min, 5:95 V/V at 11.0 min, 5:95 V/V at 12.0 min, 95:5 V/V at 12.1 min, 95:5 V/V at 15.0 min; (4) flow rate, 0.40 mL/min; (5) temperature, 40 °C; and (6) injection volume: 2 µL. The effluent was connected to an ESI-triple quadrupole-linear ion trap (Q TRAP)-MS.

The MS conditions mainly included: electrospray ionization temperature, 500 °C; mass spectrum voltage, 5500 V; curtain gas, 25 psi; collision-activated dissociation, high. In the triple quadrupole, each ion pair was scanned according to the optimized de-clustering potential and collision energy [45].

### Metabolomic analysis

Based on the Metware database and the public database of metabolite information, the material was characterized according to the secondary spectrum information. The quantification of metabolites was accomplished using a multiple reaction monitoring analysis by triple quadrupole mass spectrometry and the Analyst 1.6 software (AB Sciex) to process mass spectrometry data. According to the metabolite retention time and peak type, the mass spectrum peaks detected in different samples of each metabolite were corrected to ensure the accuracy of qualitative and quantitative data. A principal component analysis and partial least squares-discriminant analysis (PLS-DA) were applied to the data. Moreover, a hierarchical cluster analysis was performed with R (www.r-project.org/) to visualize changes in metabolic profiles.

With a fold change > 2 or < 0.5, the PLS-DA model indicated a variable importance in project (VIP) value > 1 as the threshold to screen the significantly changed metabolites between SB-0 and SB-3.

### RNA extraction, RNA-sequencing (RNA-seq), and differential expression analysis

The pericarps of SB-0 and SB-3 fruit were collected for RNA extraction. Three biological replicates were designed for each treatment. RNA was isolated from pomegranate pericarps using an RNAprep Pure Plant kit (Tiangen, China) according to the manufacturer’s protocol. Sequencing libraries were generated using the NEBNext Ultra™ RNA Library Prep Kit for Illumina (New England Biolabs, USA) following the manufacturer’s instructions. After passing the library inspection, the Illumina HiSeq platform was used for sequencing. Unigene expression levels were calculated and normalized to fragments per kilobase of transcript per million mapped reads (FPKM) [46]. Adaptor sequences and low-quality sequence reads (identity ≥ 95% and coverage length ≥ 100 bp) were removed. Furthermore, all differentially expressed genes (DEGs) were analyzed by gene ontology (GO, http://www.geneontology.org/) and Kyoto Encyclopedia of Genes and Genomes enrichment analyses (KEGG, http://www.genome.jp/kegg) [47]. The correlation matrix between DEGs assigned to significantly enriched pathways (metabolic pathways, biosynthesis of secondary metabolites, phenylpropanoid biosynthesis, and flavonoid biosynthesis) and physiological data (total phenols, total flavonoids, relative conductivity, MDA, FRAP) was generated using Pearson's correlation coefficient. Correlation matrices were visualized using the R corrplot package (https://github.com/taiyun/corrplot).

### Integrated transcriptome and metabolome analysis

Based on the gene expression and the metabolite content data, Pearson correlation tests were used to detect associations between DEGs and DAMs. The detected associations with a *P* < 0.05 were selected. In addition, DEGs and DAMs were mapped to the KEGG pathway database to obtain information about their common pathways. A co-expression analysis was conducted on genes involved in the phenylpropanoid and flavonoid biosynthesis pathways and on differentially expressed transcription factors (TFs) to identify TFs involved in the regulation of target gene expression. These analyses were performed using Cytoscape 3.7.1 (https://cytoscape.org/).

### Quantitative real-time PCR (qRT-PCR) analysis

To verify the accuracy of sequencing results, we evaluated the relative expression patterns of nine DEGs in the transcriptome data. The cDNA was synthesized using a cDNA Synthesis Kit (TaKaRa, Japan; http://ww.takara-bio.com/) following the manufacturer’s instructions. Primer Premier 5.0 software (PREMIER Biosoft International, CA, USA) was used to design gene-specific primer pairs. Information on the relevant genes and primer sequences is provided in Supplementary Table 5. The relative level of gene expression was calculated using the 2^−△△CT^ method [48]. GraphPad Prism 5 (GraphPad Software Inc., San Diego, CA, USA) was used for chart preparation. Three biological and technical replicates were performed in qRT-PCR assays. Statistical analysis of variance followed by Duncan’s new multiple range tests were performed with SPSS Version 16.0 (Chicago, IL, USA). Differences were considered significant at *P* < 0.05.

## Supplementary Information

**Additional file 1:** **Supplementary Table 1.** Differentially accumulated metabolites in sunburned compared with healthy pomegranate pericarps.

**Additional file 2:** **Supplementary Table 2.** The transcriptome sequencing results of control and sunburned pomegranate fruits.

**Additional file 3:** **Supplementary Table 3.** Differentially expressed genes in the control and sunburned pomegranate pericarps.

**Additional file 4:** **Supplementary Table 4.** Quantity and function of differently expressed transcription factors between healthy and sunburned pomegranate pericarps.

**Additional file 5:** **Supplementary Table 5.** Primers designed for the qRT-PCR expression analysis.

## Data Availability

All raw sequence data are available at NCBI project PRJNA752829 and Sequence Read Archive (SRA) with accession number SRR15412367, SRR15412368, SRR15412369, SRR15412370, SRR15412371, and SRR15412372. The addresses are as follows: https://www.ncbi.nlm.nih.gov/Traces/study/?acc=PRJNA752829. All the data and materials can be shared by contacting the corresponding author, Gaihua Qin (qghahstu@163.com).

## Abbreviations

ROS: Reactive oxygen species
AsA: Ascorbic acid
GSH: Glutathione
MDA: Malondialdehyde
TBA: Thiobarbituric acid
FRAP: Antioxidant activity
LC–MS/MS: Liquid chromatography-tandem mass spectrometry
QC: Quality control samples
PLS-DA: Partial least squares-discriminant analysis
FPKM: Fragments per kilobase of transcript per million mapped reads
DEGs: Differentially expressed genes
KEGG: Kyoto encyclopedia of genes and genomes enrichment analyses
DAMs: Differentially accumulated metabolites
TFs: Transcription factors
CAD: Cinnamyl-alcohol dehydrogenase
FLS: Flavonol synthase
ANR: Anthocyanidin reductase
HCT: Shikimate O-hydroxycinnamoyl transferase
CHS: Chalcone synthase
CHI: Chalcone isomerase
F3′5′H: Flavonoid 3′,5′-hydroxylase
